# Variation in the Use of Resection for Colorectal Cancer Liver Metastases

**DOI:** 10.1097/SLA.0000000000003534

**Published:** 2019-09-16

**Authors:** Hayley M. Fenton, John C. Taylor, J. Peter A. Lodge, Giles J. Toogood, Paul J. Finan, Alastair L. Young, Eva J. A. Morris

**Affiliations:** ∗ Cancer Epidemiology Group, Leeds Institute for Data Analytics, University of Leeds, Leeds, UK; † Department of Hepatobiliary and Transplant Surgery, St James's University Hospital, Leeds, UK.

**Keywords:** colorectal cancer, liver metastases, liver resection

## Abstract

The aim of this study was to investigate variation in the frequency of resections for colorectal cancer liver metastases across the English NHS.

**Background:**

Previous research has shown significant variation in access to liver resection surgery across the English NHS. This study uses more recent data to identify whether inequalities in access to liver resection still persist.

**Methods:**

All adults who underwent a major resection for colorectal cancer in an NHS hospital between 2005 and 2012 were identified in the COloRECTal cancer data Repository (CORECT-R). All episodes of care, occurring within 3 years of the initial bowel operation, corresponding to liver resection were identified.

**Result:**

During the study period 157,383 patients were identified as undergoing major resection for a colorectal tumor, of whom 7423 (4.7%) underwent ≥1 liver resections. The resection rate increased from 4.1% in 2005, reaching a plateau around 5% by 2012. There was significant variation in the rate of liver resection across hospitals (2.1%–12.2%). Patients with synchronous metastases who have their primary colorectal resection in a hospital with an onsite specialist hepatobiliary team were more likely to receive a liver resection (odds ratio 1.22; 95% confidence interval, 1.10–1.35) than those treated in one without. This effect was absent in resection for metachronous metastases.

**Conclusions:**

This study presents the largest reported population-based analysis of liver resection rates in colorectal cancer patients. Significant variation has been observed in patient and hospital characteristics and the likelihood of patients receiving a liver resection, with the data showing that proximity to a liver resection service is as important a factor as deprivation.

Colorectal cancer (CRC) is a common disease in the UK,^1^ and prognosis is poor if, at diagnosis, the disease has metastasized. Unfortunately, this is all too common with approximately 20% of the 42,000 people diagnosed annually^1^ having metastatic disease at presentation^2–4^ and up to 50% subsequently going on to develop it during the course of their illness.^5^ UK outcomes from CRC are known to lag behind many economically comparable countries^6^ and as this may, at least in part, be because of poorer outcomes for individuals who present with late stage disease, optimizing their care is a priority. In England, of the 35,000 people diagnosed with CRC, <60% undergo surgical resection of the primary tumor: about 21,000 cases per annum.

It is estimated that at least 15% to 20% of patients with metastases in the liver may be eligible for potentially curative liver resection.^7–9^ The National Institute of Health and Clinical Excellence guidelines recommend liver resection as the treatment of choice for metastatic disease in patients where this is possible and 10-year survival following such operations is almost 25%.^10^ Unfortunately, previous studies^11–13^ have shown that the rates of resection for CRC liver metastases (CRCLM) vary significantly across the English National Health Service (NHS) indicating there may be considerable variation in the decision-making process as to who is, and is not, eligible for resection and who is referred to a specialist liver team.^12^ The majority of the available evidence dates from >10 years ago so more contemporary data are urgently required to address the persistent concern that there may still be some patients being denied access to a specialist liver services and potentially curative treatments.^13^

This population-based study aims, therefore, to provide this information by investigating the frequency of surgical resections for CRCLM across the English NHS using the most recently available data. Trends are examined in relation to patient and tumor characteristics to identify whether the trends in likelihood of resection in relation to these factors and hospital of treatment still persist.

## METHODS

### Study Population and Data Definitions

All adults diagnosed with a first primary CRC [*International Classification of Disease* (*ICD)-10 codes C18-C20*], and who had undergone a major resection for their disease in an NHS hospital with a CRC multidisciplinary team (MDT) between January 1, 2005 and December 31, 2012 (to allow 3 years of follow-up until censoring at December 2015), were identified in the COloRECTal cancer data Repository (CORECT-R). This population-based resource^14^ contains numerous linked datasets relevant to CRC. For this study, information was derived from a linked National Cancer Registration and Analysis Service and Hospital Episode Statistics (HES) dataset. Information on date of diagnosis, age, sex, deprivation [measured via the income domain of the Index of Multiple Deprivation (IMD) 2010], site of tumor, and stage were extracted from the cancer registry dataset. Where patients had >1 tumor recorded simultaneously, the tumor with the highest stage was selected. Any remaining duplicate patient records were cleaned to select the most relevant tumor for the type of major resection carried out. Information on the type and date of the first major resection surgery following diagnosis was extracted from the HES component of CORECT-R. Major primary resection and liver resection were identified by the appropriate OPCS4.8 codes (Table 1). Primary tumors in the cecum, appendix, ascending colon, hepatic flexure, and transverse colon (*ICD*-*10 codes C18.0-C18.4*) were assigned as right-sided colon tumors, whereas tumors in the splenic flexure, descending colon and sigmoid colon (*ICD-10 C18.5-C18.7*) were assigned as left-sided colon tumors. Tumors in the rectosigmoid (*ICD-10 C19*) were classified separately because of different characteristics, as were rectal tumors (*ICD-10 C20*).

A Charlson comorbidity index^15^ was derived for each individual in the cohort, taking into account diagnoses (excluding cancer) from any hospital admissions in the year before CRC diagnosis. The cancer component of the Charlson index was derived from the cancer registry information found in CORECT-R and the score for any other cancers in the year before CRC was added to that obtained from HES data. The Charlson score was categorized as: 0, 1, 2, and ≥3 with higher scores indicating greater comorbidity.

In the English NHS, a Trust is an organization, comprising ≥1 hospitals that provide care to patients in a city or region. In this article, we have used the term “hospital” in place of Trust. Data regarding whether the initial colorectal resection was carried out within a hospital with an onsite hepatobiliary team (HBT) were obtained from the Organizational Survey 2016, carried out by the National Bowel Cancer Audit (NBOCA).^16^ As the survey is a snapshot, carried out at a date outside of the range of this dataset, the location of HBTs may change with service redesign. The survey results were checked against activity in the HES data. Liver center status was retained for hospitals where >80% of liver resections were carried out in the same hospital as patients’ colorectal primary resection, and where hepatic resection activity was consistent across the whole study period. This provided good agreement with the Organizational Survey.

### Primary Endpoints

The rate of liver resection was compared across NHS hospitals, to ascertain whether there is significant variation in the surgical management of CRC liver metastases between different providers. Analyses were undertaken with and without adjustment for casemix.

### Statistical Methods

The frequency of liver resections was assessed in relation to the year of CRC resection, patient age, sex, site and stage of the primary tumor at diagnosis, IMD quintile, Charlson score, region (based on the Cancer Alliance or Vanguard that the hospital resides in), and the hospital where the initial colorectal resection took place. The statistical significance of any differences in liver resection rates across patient characteristics and between hospitals were assessed using the chi-square test.

Multilevel binary logistic regression was used to determine factors associated with the use of resection for CRCLM, with a hierarchy of patients (level 1) clustered within hospitals (level 2), clustered further into regional Cancer Alliances (level 3). Explanatory variables in the risk-adjusted model were age at resection, sex, IMD quintile, primary tumor site, year of primary major colorectal resection, Charlson comorbidity score, stage at diagnosis, and whether the hospital was a liver center. Regression analysis was also repeated for those diagnosed with synchronous metastatic disease (Stage IV at diagnosis) and those who developed metachronous metastatic disease (Stages I-III at diagnosis). Funnel plots were constructed to show the variation across hospitals and Cancer Alliances using the Spiegelhalter approach^17^ and those hospitals or Alliances outside of the 99.8% control limits were considered outliers.

## RESULTS

### Study Population

During the period January 1, 2005 to December 31, 2012, 157,383 patients were identified as undergoing major resection for a colorectal tumor, the characteristics of which are outlined in Table 2. Of these 7423 (4.7%) underwent ≥1 liver resections within 3 years of their primary colorectal resection.

There were 117 patients who had 2 liver resections within 90 days of each other, suggesting a planned 2-stage liver resection. These are therefore treated as 1 resection episode. There were 750 patients who suffered disease recurrence requiring a further resection within the 3-year period. Overall, there were 6673 patients (89.9%) having 1 resection episode, 692 (9.3%) having 2 resection episodes, and 58 (0.8%) having ≥3, giving a total of 8234 liver resection episodes.

Most patients (6708, 90.4%) received their first liver resection after their primary colorectal resection, 499 (6.7%) had a synchronous liver resection with their primary colorectal resection and a small minority (216, 2.9%) had their first liver resection carried out before their major colorectal resection. Overall, 2236 (18.9%) of patients who presented with synchronous metastases, received a liver resection within 3 years of their primary colorectal resection. Median time from primary colorectal resection to first liver resection was 270 days.

### Variation in Resection Rates Over Time

**FIGURE 1:**
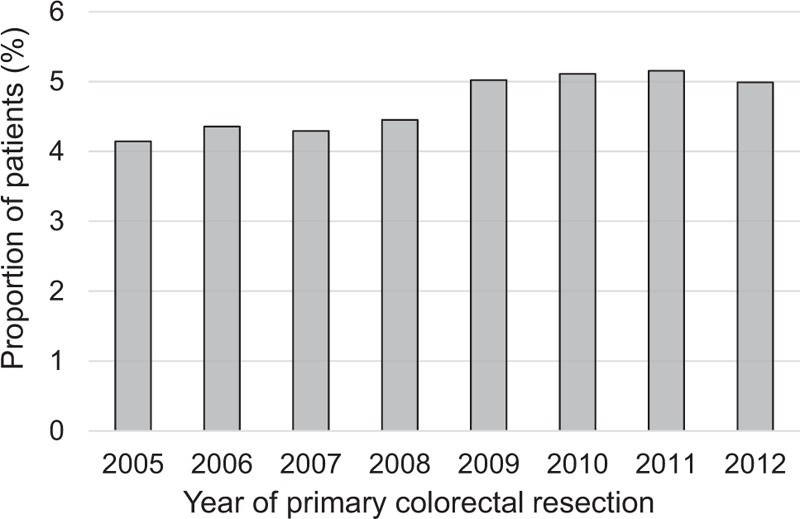
Percentage of patients who receive a major resection for colorectal cancer, and who go on to have a hepatic resection, by the year of the primary colorectal resection.

The percentage of patients receiving liver resection increased between 2005 and 2012 and appeared to have reached a stable figure of around 5% for all patients who underwent a major resection for CRC during the last 4 years of the study period (Fig. 1). The Charlson comorbidity index of the overall study population increased over time with the proportion of patients with ≥1 comorbidity increasing from 22.8% to 25.2% during the course of this study period.

The data suggest that the proportion of liver resections carried out that were classified as major resections (hemihepatectomies and extended hemihepatectomies), reduced over the study period from 48.2% to 39.9% (*P* < 0.001, Supplementary Table 1).

### Variation in Resection Rates Across Providers

**FIGURE 2:**
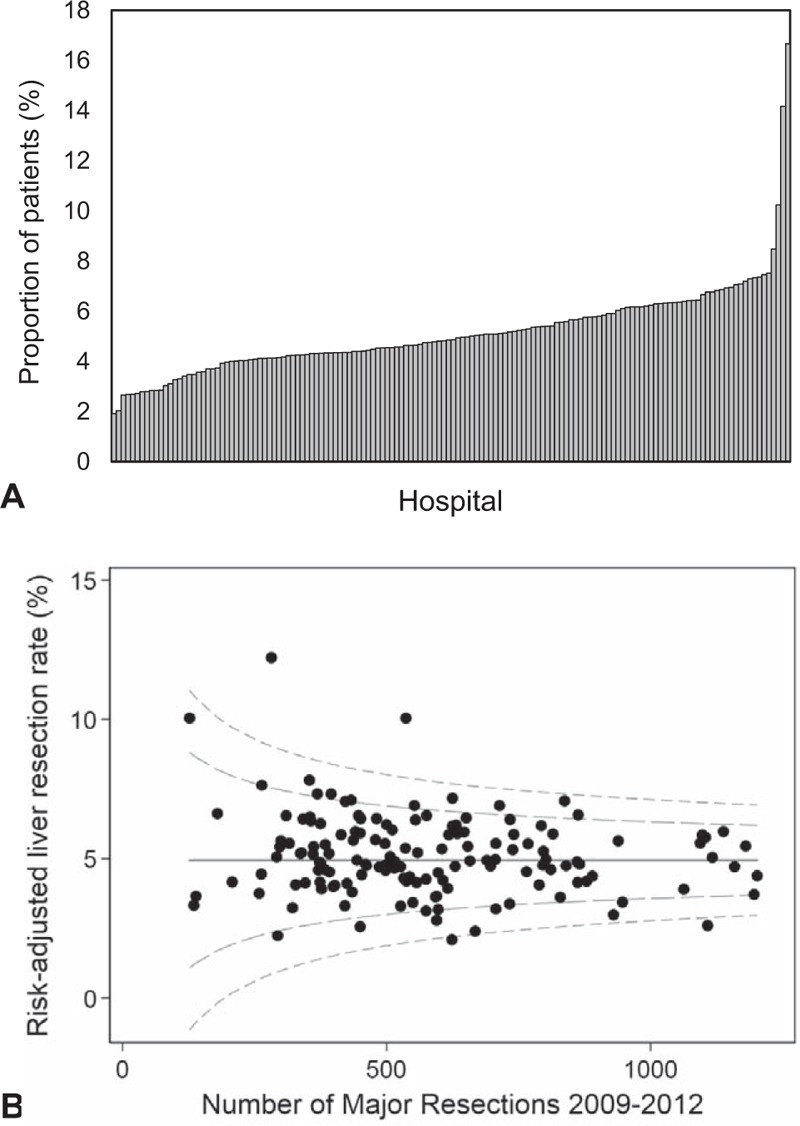
Variation in the proportion of patients receiving a liver resection within three years, by the hospital carrying out their primary colorectal resection, (A) crude rate, (B) risk-adjusted funnel plot.

The bar chart and organizational funnel plot (Fig. 2) show the variation in the rate of hepatic resection between different hospitals for the more recent time period, 2009 to 2012. There was a large degree of variation in the proportion of patients having a liver resection by the hospital where their primary tumor was treated, suggesting a difference in referral pathways between hospitals, with crude rates between 1.9% and 16.7% (*P* ≤ 0.001, Fig. 2A) and risk-adjusted rates ranging from 2.1% to 12.2% (Fig. 2B). Four hospitals were outliers (falling outside the 99.8% control limits on the funnel plot) with 2 having higher than expected rates of liver resection, and 2 having lower than expected rates. By Cancer Alliance (Supplementary Fig. 1), the crude rate ranged from 4.2% to 7.1% (*P* ≤ 0.001), with a similar risk-adjusted rate of 4.1% to –7.0%.

Analysis by the regional Cancer Alliance, within which the patient is treated for their primary colorectal resection shows that the rate of major liver resection (hemihepatectomy and extended hemihepatectomy) varied between 28% and 58% of all liver resections carried out (*P* < 0.001). This suggests there are regional differences in the surgical treatment of liver metastases.

There are also differences in the type of liver resection carried out, depending on whether patients had their primary colorectal tumor removed at a hospital with a specialist liver center onsite. The major liver resection rate was 36.1% for liver centers compared to 41.1% for those treated for their colorectal primary in a hospital without a liver centre (*P* < 0.001)

### Variation in Resection Rates by Patient Characteristics

Table 3 shows the results of the logistic regression model used to determine the odds of liver resection. There was no significant difference in the rate by year of primary resection in the adjusted model. The likelihood of liver resection decreased significantly with increasing age [odds ratio (OR) per 10-year increase 0.64; 95% confidence interval (CI), 0.62–0.65], with increasing deprivation level (OR for most deprived quintile compared to most affluent 0.76; 95% CI, 0.70–0.83)) and with increasing Charlson co-morbidity score (OR for Charlson score ≥3 compared to score of zero 0.43; 95% CI, 0.34–0.53). Women were less likely to receive a resection (OR 0.77; 95% CI, 0.73–0.81).

The odds of resection were higher for tumors in the rectosigmoid (OR 1.90; 95% CI, 1.73–2.08), rectum (OR 1.60; 95% CI, 1.49–1.71) and left colon (OR 1.66; 95% CI, 1.56–1.77) compared to the right colon. The odds also increased with advancing stage of the disease at presentation (OR Stage IV vs Stage I 20.14; 95% CI, 17.60–23.04).

In addition to the patient characteristics listed, elements of the treatment pathway affected the likelihood of resection. Patients who have their primary colorectal resection in a hospital with a liver center were more likely to receive a liver resection (OR 1.22; 95% CI; 1.10–1.35). This may be because of a higher proportion of liver resections taking place synchronously to their bowel resection for these patients (18%) compared to those receiving their bowel resection in a hospital without a specialist onsite liver center (3%).

When the logistic regression model was run to only include patients at Stages I–III at diagnosis, this effect was observed in the unadjusted model (Supplementary Table 3, OR 1.10; 95% CI ,1.02–1.19) but was no longer significant following risk-adjustment, and was only seen for those patients with synchronous metastases (Supplementary Table 2).

## DISCUSSION

This study presents the most up-to-date and largest reported population-based analysis of liver resection rates in CRC patients since Morris et al.^11^ It includes resection for both synchronous and metachronous metastatic disease and covers all patients receiving a major primary resection for CRC within the English NHS.

Despite a plateau being observed in the percentage of CRC patients undergoing a liver resection, significant differences in the likelihood of liver resection continue to be observed for different patient characteristics, hospitals, and Cancer Alliances. Women were less likely to receive a liver resection than men and this was independent of tumor location, stage, age, comorbidity, and deprivation quintile. Although the models used are not directly comparable, this reflects the findings of the previous analysis^11^ showing this trend has changed little over time. It has also been confirmed in several other studies.^11,13,18^ The lower likelihood of liver resection for women was present for all disease stages except for presentation at Stage I and so potential explanations such as differing surveillance or referral patterns between men and women should be explored in more detail. Older patients were still less likely to receive a liver resection,^11,18–20^ despite data showing they can achieve good outcomes.^21^ Although patients aged >70 years made up 51.4% of those undergoing major colorectal resection, they accounted for only 29.4% of those undergoing liver resection. It is important to note, however, that in this >70s group, liver resection rates have more than doubled over time, from 1.2% in 1998 to 2004 to 2.8% in 2005 to 2012 suggesting management patterns may have changed. The rate of major liver resection decreased over the period, from 48.2% to 33.8% of all liver resections carried out.

Increasing deprivation was also associated with a reduced likelihood of receiving a liver resection^11,20^, with those in the most deprived quintile being 24% less likely to receive a resection than those in the most affluent quintile, as was increasing comorbidity.^11,18^ In addition, these data demonstrate that anatomical location of the CRC may influence the likelihood of liver resection as the odds of receiving a resection was lowest for right-sided primary tumors. Such an observation was also noted in 2 Swedish studies.^2,18^ There has also been a change in the management of patients with synchronous metastases (Stage IV at presentation), with the proportion of these who receive liver resection surgery >doubling from 8.0% for the period 1998 to 2004 to 18.9% in 2005 to 2012.^11^

Patients who received their major primary colorectal resection in a hospital designated in this study as a liver center were 22% more likely to receive a liver resection compared to those who had to be referred on elsewhere, suggesting that referral pathways may not be working as efficiently as they should be and patients are missing out on access to treatment depending on their proximity to a hospital with a liver center. This replicates findings from a study using HES data, which examined 4547 patients who underwent major bowel resection and had synchronous metastases at diagnosis; patients treated in a hospital with an onsite hepatobiliary center were more likely to receive a liver resection.^13^ Our study confirmed this in a population of people with both synchronous and metachronous metastases and found that the increased likelihood of liver resection for patients receiving their bowel resection in a hospital with an onsite specialist liver team was present for patients at Stages I–III at diagnosis in the nonadjusted model, but this was no longer significant following risk-adjustment. However patients with synchronous metastases were 43% more likely to receive a liver resection if they had their primary tumor resected in a hospital with an onsite liver center. Numerous studies have now shown that both surgeon and hospital specialization may be associated with improved surgical outcomes, especially for complex procedures.^22–26^ This study is an important contribution to that evidence base as, across the whole population, it suggests that proximity to a liver center is as important a factor as socioeconomic deprivation when it comes to the likelihood of receiving a liver resection.

Around 35% of patients with Stage IV disease treated in a liver center received their liver resection before or at the same time as their major bowel resection, compared to 11% of patients in nonliver centers, showing a large degree of variation in the treatment pathway for synchronous metastases. Streamlining the referral process may lead to more patients being eligible for synchronous resection, which may improve outcomes.

Although linkage of the cancer registry and HES data allows full coverage of all CRC patients, there are some limitations of the study. The routine nature of the data means that there is no information as to extent of liver disease, chemotherapy status, treatment intent, or patient choice so we do not know which patients were potentially eligible for liver resection but did not undergo surgery. However, it is unlikely that the burden of liver disease varies substantially across hospitals, once adjustment for other patient and tumor characteristics is performed and so the variation highlighted remains valid.

In conclusion, a large degree of variation is still present in the rates of resection by hospital, even allowing for differences in age, stage, deprivation quintile, Charlson, tumor site, comorbidity index, year of primary resection, and whether the hospital has a liver center. This suggests that there is still variation in access to treatment for patients with both synchronous and metachronous hepatic disease and, in light of the poorer outcomes reported within the UK, such variation deserves further urgent investigation.

Despite a plateau in liver resection rates, clinicians should not become complacent in the management of CRCLM patients. Significant variations have been observed in patient and hospital characteristics and the likelihood of patients receiving a liver resection. Likely there are multiple factors influencing this; however, further work is needed to elucidate the reasons for this variation, as addressing this has a significant potential to improve patient outcomes.

## Supplementary Material

Supplemental Digital Content

## Figures and Tables

**TABLE 1:** OPCS 4.8 Codes for Major Colorectal Resection and Liver Resection

OPCS Code	Description
Primary colorectal resection	
H04	Total excision of colon and rectum
H05	Total excision of colon
H06	Extended excision of right hemicolon
H07	Other excision of right hemicolon
H08	Excision of transverse colon
H09	Excision of left hemicolon
H10	Excision of sigmoid colon
H11	Other excision of colon
H29	Subtotal excision of colon
H33	Excision of rectum
X14	Clearance of pelvis
Liver resection	
J021	Right hemihepatectomy NEC
J022	Left hemihepatectomy NEC
J023	Resection of segment of liver
J024	Wedge excision of liver
J026	Extended right hemihepatectomy
J027	Extended left hemihepatectomy
J028	Other specified partial excision of liver
J029	Partial excision of liver unspecified
J031	Excision of lesion of liver
J035	Excision of multiple lesions of liver
J038	Other specified extirpation of lesion of liver
J039	Unspecified extirpation of lesion of liver

**TABLE 2:** Characteristics of the Study Population

	No Resection (n = 149,960)	Liver Resection (n = 7423)	Total (n = 157,383)
Tumor site			
Colon	101,133 (67.4)	4584 (61.8)	105,717 (67.2)
Cecum	24,307 (16.2)	846 (11.4)	25,153 (16)
Appendix	1263 (0.8)	42 (0.6)	1305 (0.8)
Ascending colon	14,585 (9.7)	481 (6.5)	15,066 (9.6)
Hepatic flexure	4724 (3.2)	208 (2.8)	4932 (3.1)
Transverse colon	8709 (5.8)	306 (4.1)	9015 (5.7)
Splenic flexure	3640 (2.4)	189 (2.5)	3829 (2.4)
Descending colon	4623 (3.1)	228 (3.1)	4851 (3.1)
Sigmoid colon	33,601 (22.4)	2078 (28)	35,679 (22.7)
Overlapping/unspecified lesion of colon	5681 (3.8)	206 (2.8)	5887 (3.7)
Rectosigmoid	10,786 (7.2)	730 (9.8)	11,516 (7.3)
Rectum	38,041 (25.4)	2109 (28.4)	40,150 (25.5)
Sex			
Male	83,603 (55.8)	4660 (62.8)	88,263 (56.1)
Female	66,357 (44.2)	2763 (37.2)	69,120 (43.9)
Age at primary colorectal resection			
≤60	28,511 (19)	2466 (33.2)	30,977 (19.7)
61–70	42,715 (28.5)	2777 (37.4)	45,492 (28.9)
71–80	51,157 (34.1)	1950 (26.3)	53,107 (33.7)
>80	27,577 (18.4)	230 (3.1)	27,807 (17.7)
Year of primary colorectal resection			
2005	15,773 (10.5)	682 (9.2)	16,455 (10.5)
2006	17,676 (11.8)	805 (10.8)	18,481 (11.7)
2007	18,237 (12.2)	818 (11)	19,055 (12.1)
2008	19,116 (12.7)	891 (12)	20,007 (12.7)
2009	19,211 (12.8)	1016 (13.7)	20,227 (12.9)
2010	19,842 (13.2)	1069 (14.4)	20,911 (13.3)
2011	19,951 (13.3)	1084 (14.6)	21,035 (13.4)
2012	20,154 (13.4)	1058 (14.3)	21,212 (13.5)
Tumor stage at diagnosis			
I	19,279 (12.9)	252 (3.4)	19,531 (12.4)
II	49,202 (32.8)	1397 (18.8)	50,599 (32.2)
III	47,330 (31.6)	2509 (33.8)	49,839 (31.7)
IV	9587 (6.4)	2236 (30.1)	11,823 (7.5)
Unknown	24,562 (16.4)	1029 (13.9)	25,591 (16.3)
IMD quintile			
1—least deprived	33,480 (22.3)	1820 (24.5)	35,300 (22.4)
2	34,022 (22.7)	1755 (23.6)	35,777 (22.7)
3	31,763 (21.2)	1528 (20.6)	33,291 (21.2)
4	27,886 (18.6)	1308 (17.6)	29,194 (18.5)
5—most deprived	22,809 (15.2)	1012 (13.6)	23,821 (15.1)
Charlson Comorbidity Index			
0	111,599 (74.4)	6099 (82.2)	117,698 (74.8)
1	25,917 (17.3)	1031 (13.9)	26,948 (17.1)
2	7668 (5.1)	215 (2.9)	7883 (5.0)
≥3	4776 (3.2)	78 (1.1)	4854 (3.1)

Values in parentheses are percentages.

**TABLE 3:** Odds of Having a Liver Resection Within 3 Years of Primary Colorectal Tumor Resection

	Unadjusted Odds Ratio	95% Confidence Interval		*P*	*P* Across Groups	Adjusted Odds Ratio	95% Confidence Interval		*P*	*P* Across Groups
Year of resection of colorectal primary	1.03	1.02	1.05	<0.001	<0.001	1.00	1.00	1.02	0.155	0.155
Primary resection carried out in hospital with liver center	1.29	1.23	1.37	<0.001	<0.001	1.22	1.10	1.35	<0.001	<0.001
Age at resection of colorectal primary (per 10-y increase)	0.58	0.56	0.59	<0.001	<0.001	0.63	0.62	0.65	<0.001	<0.001
Sex					<0.001					<0.001
Male	1.00					1.00				
Female	0.75	0.71	0.78	<0.001		0.77	0.73	0.81	<0.001	
IMD quintile					<0.001					<0.001
1—least deprived	1.00					1.00				
2	0.95	0.89	1.01	0.127		0.97	0.90	1.03	0.307	
3	0.88	0.83	0.95	0.001		0.90	0.83	0.97	0.004	
4	0.86	0.80	0.93	<0.001		0.87	0.81	0.94	<0.001	
5—most deprived	0.82	0.75	0.88	<0.001		0.76	0.70	0.83	<0.001	
Stage of primary tumor at diagnosis					<0.001					<0.001
I	1.00					1.00				
II	2.17	1.90	2.49	<0.001		2.66	2.32	3.04	<0.001	
III	4.06	3.56	4.62	<0.001		4.46	3.91	5.09	<0.001	
IV	17.84	15.63	20.37	<0.001		20.14	17.60	23.04	<0.001	
unknown	3.21	2.79	3.68	<0.001		2.97	2.57	3.43	<0.001	
Tumour site					<0.001					<0.001
Right colon	1.00					1.00				
Left colon	1.70	1.60	1.80	<0.001		1.66	1.56	1.77	<0.001	
Rectosigmoid	1.92	1.76	2.10	<0.001		1.90	1.73	2.08	<0.001	
Rectum	1.58	1.48	1.68	<0.001		1.60	1.49	1.71	<0.001	
Colon unknown	1.03	0.89	1.19	0.674		1.06	0.91	1.23	0.469	
Charlson comorbidity score					<0.001					<0.001
0	1.00					1.00				
1	0.73	0.68	0.78	<0.001		0.85	0.79	0.91	<0.001	
2	0.51	0.45	0.59	<0.001		0.68	0.59	0.79	<0.001	
≥3	0.30	0.24	0.37	<0.001		0.43	0.34	0.53	<0.001	

## DISCUSSANTS

### Marek Krawczyk (Warsaw, Poland):

First of all, I would like to thank the European Surgical Association for giving me the opportunity to be the first discussant of such an interesting study.

I have to congratulate the authors on the preparation of this study. They observed significant variation in the likelihood of liver resection, with liver resection being more likely for patients who had their primary tumors resected in a hospital with a specialist hepatobiliary service.

However, a few questions arise during the evaluation of the study:

First, what was the effect in patients with complicated colon cancer (ie, perforation or bleeding) and liver metastases resected in the departments with or without liver surgery?

Second, what was the influence of the number and size of liver metastases in the departments with or without liver surgery on the survival of the patients?

Third, I’d like to know whether the chemical treatment has affected the performance of the resection of the liver with metachronic metastases. Perhaps, patients from wards that do not have liver surgery were only referred to chemotherapy without taking into account liver resection.

I would be pleased to hear your answers to my questions.

### Response From Peter A. Lodge (Leeds, United Kingdom):

Thank you for some really good questions. Obviously, one of the advantages of these datasets is the extensive population coverage. Unfortunately, with these administrative datasets, we do lose that clinical detail. We don’t know of any perforation or bleeding, and the effect that they may have. I can’t think of a population-based dataset in England, which would have that information. We are only able to identify the patients having colorectal surgery, but we’re not currently able to identify what complications they had before they had it. I think the same goes for the number, the size of the tumors, and the chemical treatment. These are clearly very important points to look at, but they are currently not recorded in the national dataset.

### Massimo Malagò (London, United Kingdom):

I have the privilege to work in the NHS and live in the United Kingdom, and I know and live the problems you mentioned in this interesting paper. Basically, it is very important that if there is an HPB-MDT or work in an environment close to an MDT, every case must be discussed at the regional HPB-MDT. Now, there is a article by Graham Poston in BJS from 2010, which also states that there are regional differences and different outcomes for HPB cancers throughout the UK. We tried to enforce the presentation of all patients with HPB cancers to specialist MDTs, but unfortunately, it seems that this does not work yet. So, what should we do to provide true equality of care in and beyond the UK?

### Response From Peter A. Lodge (Leeds, United Kingdom):

You make an important point. I think that what we’re trying to get at is that there are too many nonliver surgeons making decisions about what's operable or inoperable. Although there is an obligation for reporting the registration of primary hepatobiliary cancer in the UK, there's currently no obligation for the registration and MDT discussion of secondary CRC. Currently, I think that all we can do is go to the colorectal MDTs and encourage those physicians to send them in, unless we have very good links. However, there's always resistance from some of those groups. It's a difficult problem. I suspect that if we looked at something like recurrent CRC, we’d probably see a similar outcome. In Leeds, we have a high rate of dealing with recurrent CRC, but not all of the hospitals around will refer patients. It's an important topic, and trying to get the word out is key.

### Felice Giuliante (Rome, Italy):

Thank you very much for your extremely interesting data and analysis. First, the issue lies in the lack of availability of liver surgeons in hospitals treating patients with CRCs; second, colorectal surgeons and medical oncologists should be made aware that the evaluation of the resectability of liver metastases should be done by dedicated liver surgeons, as this can change the clinical history of these patients. Of course, this is not only a problem for UK patients, but the fact that you clearly highlighted this important issue makes your study especially interesting. Do you think that a possible solution could be to create official networks, in which there are hospitals with colorectal surgery units and medical oncology units, which would then be connected to a specialized liver surgery unit in a referral hospital? This could be achieved through regular web conferences between different hospitals, during which official cases and imaging would be presented. In the UK, where liver surgery has been strongly centralized, this could be a way to face up to this problem of inequality in care. Could you comment on this?

### Response From Peter A. Lodge (Leeds, United Kingdom):

I think that's really important. Another piece of work we’ve done recently is to analyze colorectal MDTs within our region, to see whether we have access to all of their scans and so on, and verify what they should have referred. There were about one-third of patients, who they didn’t refer and we could have operated on. For this reason, resection rates remained low. It's about education and trying to get the word out.

## Pierre-Alain Clavien (Zurich, Switzerland):

Congratulations, Professor Lodge, for this important article. England is a role model for the centralization of complex procedures, with only a few centers performing major liver surgery. There was a similar article presented at the recent American Surgical Association meeting in Dallas, which reported on geographic variation in the utilization of liver resection in California with an effect on overall survival rate in CRC. This article was published in the September 2019 issue of *Annals of Surgery*. The issue in England seems to relate to the availability of MDTs, which correlates with the use of liver resection for colorectal liver metastases. How should this be fixed? With the mission of centralization, should large expert centers take the responsibility to organize MDTs with their own specialists, rather than just work in a network, expecting proper referrals? Should the government take the lead for such MDTs and better support the large centers for such a mission? I would love to have your input on this.

### Response From Peter A. Lodge (Leeds, United Kingdom):

I think that this is a good question, but a difficult one to answer. The trouble is that there are local and national political influences. We can do our best to reach out to the MDT groups, which are not referring patients, but we have limited time and personnel. It certainly requires more investment.
