# Supplementary material for: Variation in the Use of Resection for Colorectal Cancer Liver Metastases
Source: Ann Surg. 2019 Sep 16;270(5):892–8. doi: 10.1097/SLA.0000000000003534 (PMC6867670; doi:10.1097/SLA.0000000000003534)

Supplementary Table 1: Rates of major and minor liver resections by the year of primary colorectal resection

| Year of primary colorectal resection | Minor liver resection | | Hemihepatectomy | | Extended hemihepatectomy | | Total |
| --- | --- | --- | --- | --- | --- | --- | --- |
|  | n | % | n | % | n | % |  |
| 2005 | 353 | 51.76 | 276 | 40.47 | 53 | 7.77 | 682 |
| 2006 | 436 | 54.16 | 302 | 37.52 | 67 | 8.32 | 805 |
| 2007 | 465 | 56.85 | 279 | 34.11 | 74 | 9.05 | 818 |
| 2008 | 531 | 59.60 | 275 | 30.86 | 85 | 9.54 | 891 |
| 2009 | 608 | 59.84 | 312 | 30.71 | 96 | 9.45 | 1,016 |
| 2010 | 669 | 62.58 | 318 | 29.75 | 82 | 7.67 | 1,069 |
| 2011 | 698 | 64.39 | 311 | 28.69 | 75 | 6.92 | 1,084 |
| 2012 | 700 | 66.16 | 293 | 27.69 | 65 | 6.14 | 1,058 |
|  |  |  |  |  |  |  |  |
| Total | 4,460 |  | 2,366 |  | 597 |  | 7,423 |

Supplementary Table 2: Odds of having a liver resection within 3 years of primary colorectal tumour resection for those with synchronous metastases (Stage IV at diagnosis)

|  | Unadjusted odds ratio | 95% Confidence Interval | | P | P across groups | Adjusted odds ratio | 95% Confidence | | P | P across groups |
| --- | --- | --- | --- | --- | --- | --- | --- | --- | --- | --- |
|  |  |  |  |  |  |  | Interval | |  |  |
| Year of resection of colorectal primary | 1.05 | 1.03 | 1.07 | <0.001 | <0.001 | 1.07 | 1.04 | 1.09 | <0.001 | <0.001 |
| Primary resection carried out in trust with liver centre | 1.43 | 1.29 | 1.59 | <0.001 | <0.001 | 1.43 | 1.20 | 1.70 | <0.001 | <0.001 |
| Age at resection of colorectal primary  (per 10-year increase) | 0.61 | 0.58 | 0.64 | <0.001 | <0.001 | 0.65 | 0.62 | 0.68 | <0.001 | <0.001 |
| Sex |  |  |  |  | <0.001 |  |  |  |  | <0.001 |
| Male | 1.00 |  |  |  |  | 1.00 |  |  |  |  |
| Female | 0.71 | 0.65 | 0.78 | <0.001 |  | 0.76 | 0.68 | 0.83 | <0.001 |  |
| IMD quintile |  |  |  |  | <0.001 |  |  |  |  | <0.001 |
| 1 - least deprived | 1.00 |  |  |  |  | 1.00 |  |  |  |  |
| 2 | 1.04 | 0.91 | 1.19 | 0.523 |  | 1.05 | 0.92 | 1.21 | 0.47 |  |
| 3 | 0.84 | 0.73 | 0.97 | 0.015 |  | 0.83 | 0.72 | 0.97 | 0.016 |  |
| 4 | 0.85 | 0.74 | 0.98 | 0.027 |  | 0.86 | 0.74 | 1.01 | 0.063 |  |
| 5 - most deprived | 0.73 | 0.62 | 0.85 | <0.001 |  | 0.69 | 0.58 | 0.82 | <0.001 |  |
| Tumour site |  |  |  |  | <0.001 |  |  |  |  | <0.001 |
| Right Colon | 1.00 |  |  |  |  | 1.00 |  |  |  |  |
| Left Colon | 2.10 | 1.87 | 2.36 | 0.607 |  | 1.97 | 1.74 | 2.22 | <0.001 |  |
| Rectosigmoid | 2.83 | 2.37 | 3.37 | <0.001 |  | 2.65 | 2.20 | 3.19 | <0.001 |  |
| Rectum | 3.19 | 2.80 | 3.63 | <0.001 |  | 2.64 | 2.30 | 3.02 | <0.001 |  |
| Colon unknown | 1.09 | 0.79 | 1.49 | <0.001 |  | 1.09 | 0.79 | 1.52 | 0.595 |  |
| Charlson co-morbidity score |  |  |  |  | <0.001 |  |  |  |  | <0.001 |
| 0 | 1.00 |  |  |  |  | 1.00 |  |  |  |  |
| 1 | 0.74 | 0.65 | 0.84 | <0.001 |  | 0.94 | 0.82 | 1.07 | 0.343 |  |
| 2 | 0.41 | 0.31 | 0.54 | <0.001 |  | 0.60 | 0.45 | 0.80 | <0.001 |  |
| ≥3 | 0.19 | 0.12 | 0.32 | <0.001 |  | 0.31 | 0.18 | 0.51 | <0.001 |  |

Supplementary Table 3: Odds of having a liver resection within 3 years of primary colorectal tumour resection for those with stages I-III disease at diagnosis

|  | Unadjusted odds ratio | 95% Confidence Interval | | P | P across groups | Adjusted odds ratio | 95% Confidence  Interval | | P | P across groups |
| --- | --- | --- | --- | --- | --- | --- | --- | --- | --- | --- |
| Year of resection of colorectal primary | 0.99 | 0.98 | 1.00 | 0.19 | 0.19 | 0.99 | 0.97 | 1.00 | 0.058 | 0.058 |
| Primary resection carried out in trust with liver centre | 1.10 | 1.02 | 1.19 | 0.014 | 0.014 | 1.07 | 0.97 | 1.17 | 0.199 | 0.199 |
| Age at resection of colorectal primary  (per 10-year increase) | 0.59 | 0.57 | 0.61 | <0.001 | <0.001 | 0.63 | 0.61 | 0.65 | <0.001 | <0.001 |
| Sex |  |  |  |  | <0.001 |  |  |  |  | <0.001 |
| Male | 1.00 |  |  |  |  | 1.00 |  |  |  |  |
| Female | 0.71 | 0.66 | 0.75 | <0.001 |  | 0.75 | 0.71 | 0.81 | <0.001 |  |
| IMD quintile |  |  |  |  | <0.001 |  |  |  |  | <0.001 |
| 1 - least deprived | 1.00 |  |  |  |  | 1.00 |  |  |  |  |
| 2 | 0.91 | 0.83 | 0.99 | 0.031 |  | 0.93 | 0.85 | 1.01 | 0.094 |  |
| 3 | 0.86 | 0.79 | 0.94 | 0.001 |  | 0.88 | 0.80 | 0.97 | 0.008 |  |
| 4 | 0.86 | 0.78 | 0.95 | 0.002 |  | 0.87 | 0.79 | 0.96 | 0.007 |  |
| 5 - most deprived | 0.80 | 0.72 | 0.89 | <0.001 |  | 0.76 | 0.68 | 0.85 | <0.001 |  |
| Stage of primary tumour at diagnosis |  |  |  |  | <0.001 |  |  |  |  | <0.001 |
| I | 1.00 |  |  |  |  | 1.00 |  |  |  |  |
| II | 2.17 | 1.90 | 2.49 | <0.001 |  | 2.58 | 2.25 | 2.96 | <0.001 |  |
| III | 4.06 | 3.56 | 4.62 | <0.001 |  | 4.36 | 3.82 | 4.98 | <0.001 |  |
| Tumour site |  |  |  |  | <0.001 |  |  |  |  | <0.001 |
| Right Colon | 1.00 |  |  |  |  | 1.00 |  |  |  |  |
| Left Colon | 1.73 | 1.60 | 1.88 | 0.151 |  | 1.55 | 1.43 | 1.68 | <0.001 |  |
| Rectosigmoid | 1.91 | 1.69 | 2.14 | <0.001 |  | 1.64 | 1.45 | 1.85 | <0.001 |  |
| Rectum | 1.68 | 1.54 | 1.82 | <0.001 |  | 1.45 | 1.32 | 1.58 | <0.001 |  |
| Colon unknown | 1.16 | 0.95 | 1.41 | <0.001 |  | 1.06 | 0.87 | 1.30 | 0.561 |  |
| Charlson co-morbidity score |  |  |  |  | <0.001 |  |  |  |  | <0.001 |
| 0 | 1.00 |  |  |  |  | 1.00 |  |  |  |  |
| 1 | 0.70 | 0.64 | 0.76 | <0.001 |  | 0.83 | 0.76 | 0.92 | <0.001 |  |
| 2 | 0.57 | 0.48 | 0.68 | <0.001 |  | 0.77 | 0.65 | 0.92 | 0.004 |  |
| ≥3 | 0.37 | 0.28 | 0.48 | <0.001 |  | 0.51 | 0.39 | 0.68 | <0.001 |  |

Supplementary Figure 1: Variation in the proportion of patients receiving a liver resection within three years, by the Cancer Alliance within which their primary colorectal resection takes place. A) crude rate, B) risk-adjusted funnel plot


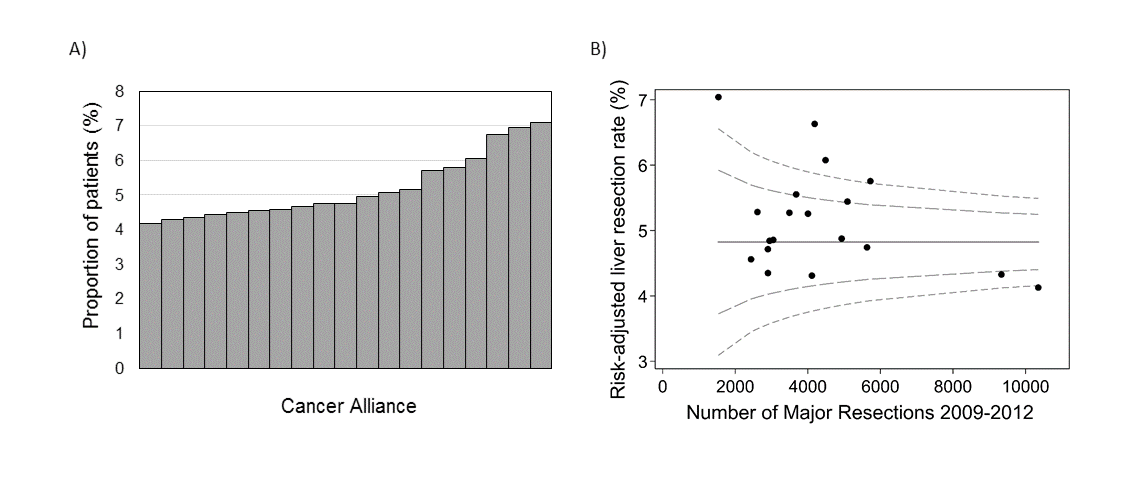

Supplement: Supplemental Digital Content [file ansu-270-892-s001.docx]
